# Monomineralic anorthosites in layered intrusions are indicators of the magma chamber replenishment by plagioclase-only-saturated melts

**DOI:** 10.1038/s41598-020-60778-w

**Published:** 2020-03-02

**Authors:** Rais Latypov, Sofya Chistyakova, Gelu Costin, Olivier Namur, Steve Barnes, Willem Kruger

**Affiliations:** 1 School of Geosciences, University of the Witwatersrand, Johannesburg, South Africa; 2 Department of Earth Science, Rice University, Houston, Texas USA; 3 Department of Earth and Environmental Sciences, KU Leuven, 3001 Leuven, Belgium; 4 CSIRO Mineral Resources, Kensington, Perth, WA 6151 Australia

**Keywords:** Geochemistry, Petrology

## Abstract

The formation of some Earth’s monomineralic igneous rocks appears to be prohibited by constraints imposed by liquidus phase-equilibria on evolution of mantle-derived magmas. Yet, these rocks exist as stratiform layers in many mafic-ultramafic intrusions. One conspicuous example is monomineralic anorthosites in the Bushveld Complex that occur as stratiform layers up to hundreds of kilometres in length. Such monomineralic anorthosites appear to require parental melts saturated in plagioclase only but where and how to produce these melts remains a contentious issue. Here we argue that they are likely sourced from deep-seated magma reservoirs. In response to pressure reduction, these ascending melts become first superheated and then saturated in plagioclase after stalling and cooling in shallow-level chambers. Adcumulus growth of plagioclase from such melts at the chamber floor results in the formation of monomineralic anorthosites. We propose that stratiform layers of monomineralic anorthosites in layered intrusions are products of the chamber replenishment by melts whose saturation in plagioclase as a single liquidus phase is triggered by their transcrustal ascent towards the Earth’s surface.

## Introduction

## A Problem of Stratiform Monomineralic Rocks in Layered Intrusions

The Earth’s mantle is rich in olivine and therefore it generates melts that, after their ascending and emplacement to a shallow level, are commonly saturated in olivine^1^ and may initially crystallize monomineralic dunite^2^; with further differentiation some of these melts may also produce monomineralic orthopyroxenite^3^. Subsequent evolution of these melts in crustal chambers results in olivine/orthopyroxene to be joined by an increasing number of crystallizing phases (up to 7–8 phases) to form various polymineralic cumulates (norite, gabbronorite, pigeonite gabbro, etc.). This general evolution of melts along multi-phase cotectics/eutectics is dictated by liquidus phase equilibria and is therefore regarded as one of the most fundamental principles of igneous petrology^4–8^. However, in many mafic-ultramafic intrusions the sequence of polymineralic cumulates is interrupted by a sudden appearance of stratiform layers of various monomineralic rocks (e.g. anorthosite, clinopyroxenite, hornblendite, chromitite and magnetitite)^8^, which are not expected to form from the multi-saturated melts. Some of these rocks have been attributed to density-related sorting of minerals in the chamber – e.g. settling of dense mafic minerals (e.g. olivine or pyroxenes) at the chamber’s floor^4,9,10^ or plagioclase flotation/suspension in the chamber^11–13^, but major problems exist with such interpretations^14–16^. A simpler way of explaining stratiform layers of monomineralic rocks appears to be their direct crystallization from single-phase-saturated melts^17^, i.e. saturated in plagioclase, clinopyroxene, hornblende, chromite or magnetite, alone.

However, at issue is how to produce such single-phase-saturated melts in the magma chamber whose resident melt is normally saturated in multiple phases. The problem can be best addressed by examining stratiform anorthosites – the most prominent monomineralic rocks that occur as continuous layers in many mafic-ultramafic intrusions^6–8,13^. The available experimental studies indicate that mantle-derived basaltic to picritic melts will have plagioclase that only appears on the liquidus as a second, third or even a fourth phase after olivine and both pyroxenes^1,18^. Therefore, it seems impossible to produce plagioclase-only-saturated liquids by fractional crystallization of primary mantle-derived melts. The major challenge is thus to understand why stratiform layers of monomineralic anorthosites, nonetheless, exist in nature despite the severe obstacles from liquidus phase equilibria. Similar challenges emerge when trying to explain other monomineralic rocks in plutonic complexes^7,8,15,19^. In this study we argue that an important clue to understanding of stratiform layers of monomineralic rocks in igneous complexes is provided by transgressive relationships of stratiform anorthosites with their footwall rocks in the Upper Critical Zone (UCZ) of the Bushveld Complex in South Africa – the largest basaltic magma chamber preserved in the Earth’s crust^19^.

## Stratiform Layers of Monomineralic Anorthosites in the Bushveld Complex

**Figure 1:**
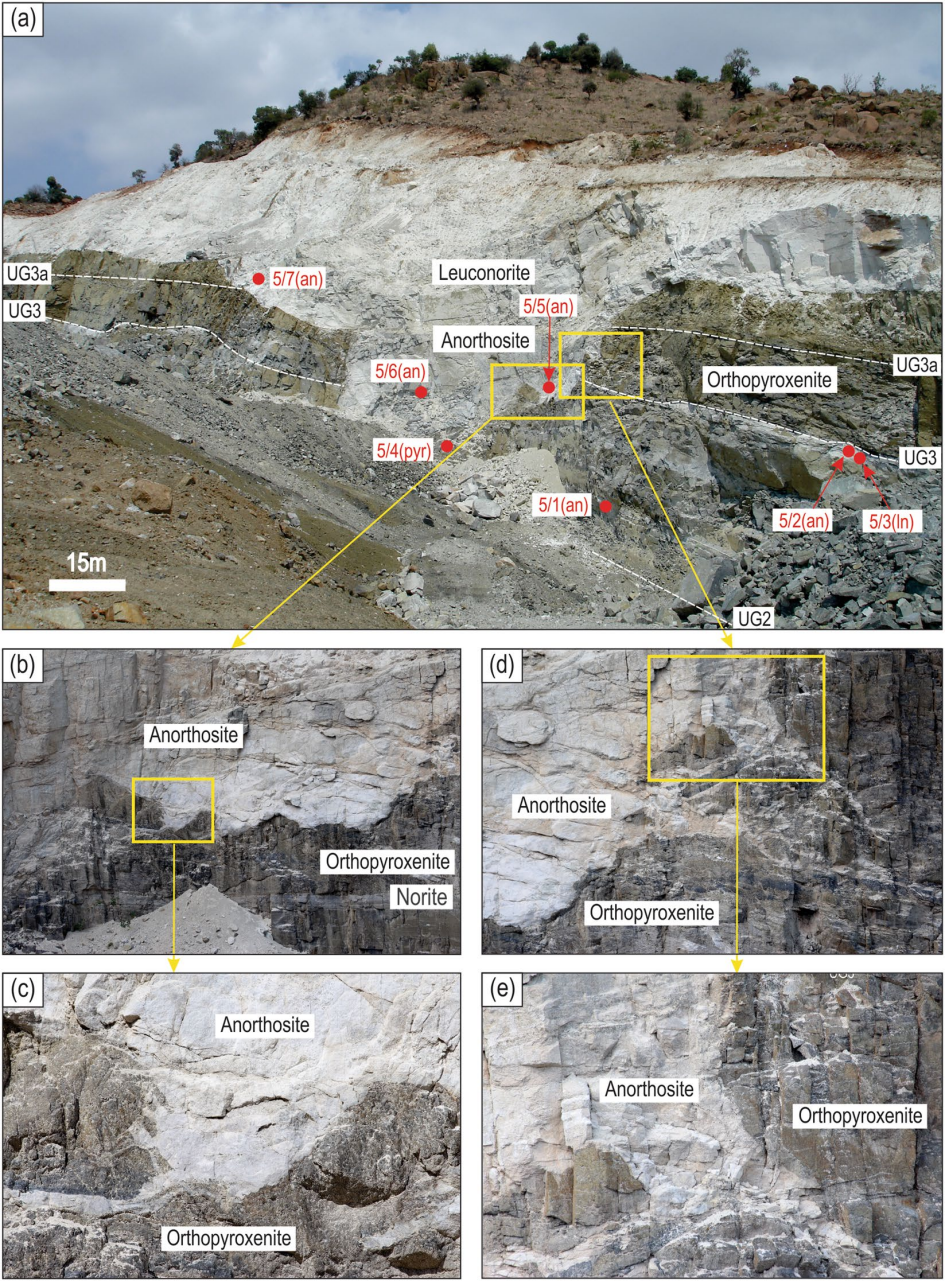
Photographs showing a large anorthosite pothole that transgresses footwall rocks at Smokey Hills mine, Eastern Bushveld. (**a)** Anorthosite/leuconorite forms a pothole cutting through the UG3/UG3a and UG2 footwall sequence composed mostly of chromitite and orthopyroxenite with some anorthosite and leuconorite sublayers **(b,c)** Close-ups showing the highly undulating and scalloped erosive contact of anorthosite with the footwall orthopyroxenite. An angular block of orthopyroxenite/chromitite occurs at the base of the pothole. **(d,e)** Close-ups of the pothole’s margin showing anorthosite protrusions and veins that invade the footwall orthopyroxenite. We interpret these field relationships as suggestive of anorthosite formation from highly reactive melts that replenished the evolving chamber and caused erosion of footwall cumulates with subsequent crystallization of plagioclase from plagioclase-only-saturated melts. Location of rock samples involved in the study is indicated in (**a**). Abbreviations: an – anorthosite; ln – leuconorite; pyr – orthopyroxenite. Photograph (**a**) is courtesy of Thomas Oberthür.

There are dozens of stratiform anorthosite layers in the UCZ of the Bushveld Complex. Spectacular outcrops have recently been documented in the UCZ of both the eastern^20^ and western^21^ Bushveld Complex where such anorthosites fill depressions (potholes) cutting down up to dozens of metres through the underlying stratigraphy. Such previously unknown relationships have only recently been revealed by large-scale open pit activities of platinum and chrome mining companies. One of the best examples illustrates a pothole ~10 m deep at Smokey Hills mine, Eastern Bushveld (Fig. 1). The pothole is filled with an anorthosite/leuconorite layer that overlies a sequence of UG2/UG3/UG3a mafic-ultramafic units (Fig. 1a; UG – Upper Group). The UG2 unit is composed of a thick chromitite layer overlain by orthopyroxenite with some norites and anorthosite sublayers. The UG3/UG3a units comprise thinner chromitite layers mostly overlain by orthopyroxenite. Immediately underlying the UG3 chromitite is a prominent anorthosite/leuconorite layer (Fig. 1a). The pothole cuts through the entire underlying sequence down to the UG2 chromitite. The transgression is well seen from the discordant relationships of UG3/UG3a chromitite and anorthosite with the sub-vertical margins of the pothole (Fig. 1a). In the pothole, the cross-cutting relationships are particularly evident from the occurrence of angular to sub-rounded blocks of footwall rocks (chromitite, orthopyroxenite, and norite) within anorthosite/leuconorite (Fig. 1b,c), from the highly undulating and scalloped nature of the basal contact of the potholed anorthosites (Fig. 1b,c) and from the local invasion of thin offshoots of anorthosite from steeply-inclined sidewalls into adjacent footwall orthopyroxenite^20^ (Fig. 1d,e). These transgressive relationships are restricted to the lower contact of the anorthosite unit and are not observed at its top surface.

**Figure 2:**
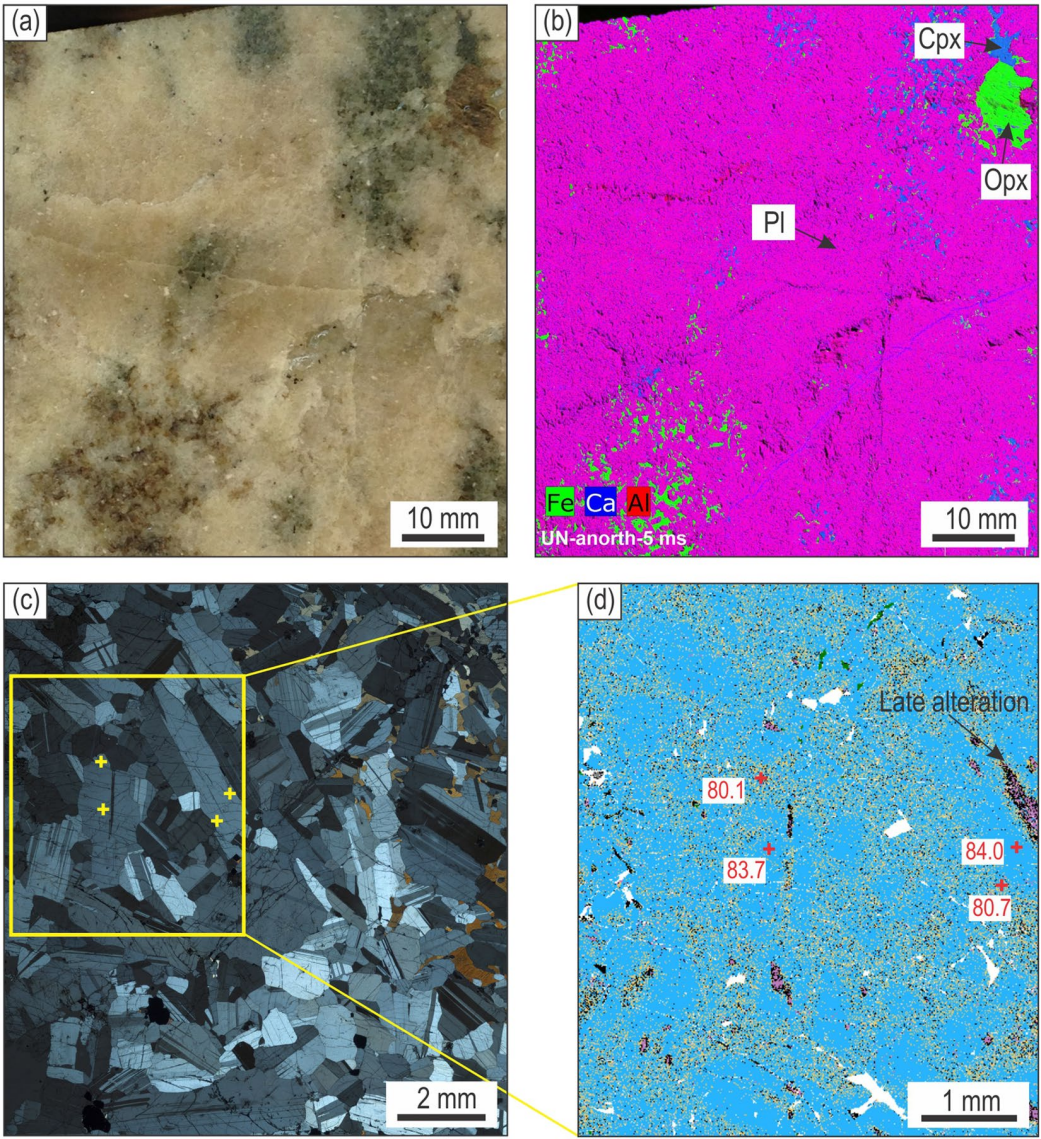
Textural and compositional features of anorthosite from the basal part of the pothole at Smokey Hills mine, Eastern Bushveld. (**a**) Photograph and (**b**) XRF image of a slab of nearly monomineralic anorthosite with dark mottles of oikocrysts of ortho- and clinopyroxene (Methods, XRF imaging of rock samples). **(c)** Photomicrograph under crossed polarized light illustrating texturally equilibrated geometry of plagioclase crystals in a highly densified anorthosite. **(d)** Ca element map showing limited chemical zonation in plagioclase crystals from a highly densified anorthosite. An-content are indicated in a few points. All figures are from sample Bu-5/5 (see Fig. 1 for its location). Modal composition, texture and a limited zonation in plagioclase indicate that Bu-5/5 anorthosite is a nearly perfect adcumulate. Abbreviations: Pl, plagioclase; Opx, orthopyroxene; Cpx, clinopyroxene.

**Figure 3:**
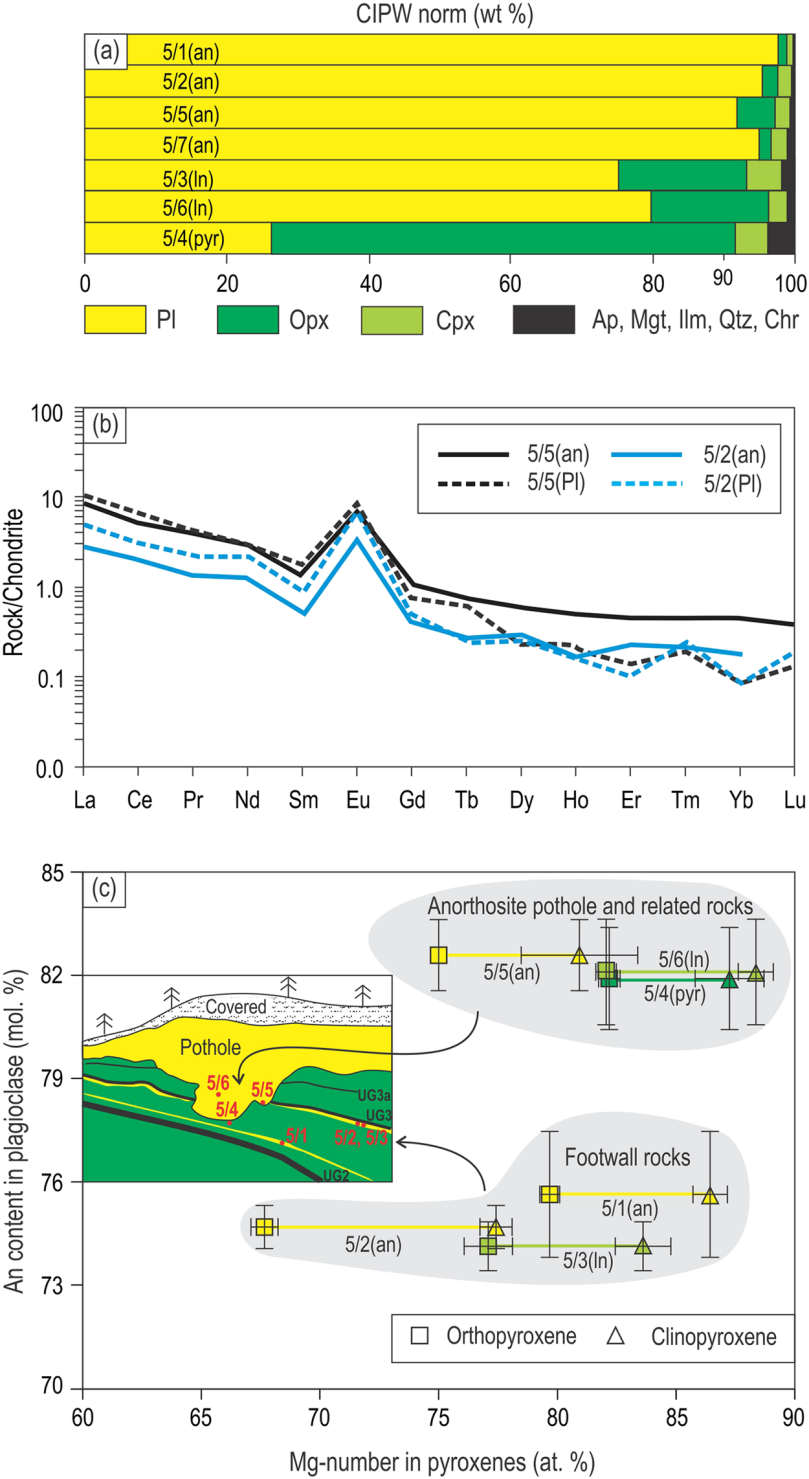
CIPW normative compositions of rocks, chondrite-normalized REE patterns of anorthosites/plagioclases and variations in plagioclase versus pyroxenes compositions in rocks from the pothole and its footwall. (**a)** Modal composition of the studied cumulate rocks based on CIPW norms. Note the nearly monomineralic composition of anorthosites (Methods, XRF and ICP-MS analyses of major and trace elements). **(b)** Similarity in chondrite-normalized REE patterns of anorthosites and their plagioclases indicative of the adcumulate nature of the anorthosites (Methods, Laser ablation inductively coupled plasma mass spectrometry). **(c)** Average An-content of plagioclase in all rocks that form or are closely associated with the pothole is higher than that of anorthosite/leuconorite from the footwall. Oikocrysts of clino- and orthopyroxene in anorthosites show average Mg-numbers that are either similar to or only slightly lower than cumulus orthopyroxenes in adjacent leuconorite/orthopyroxenite (Methods, Electron probe microanalysis of minerals). Insert in (**c**) shows the location of the studied samples relative to a pothole. The reversal towards more primitive composition of plagioclase suggests that anorthosite/leuconorite in a pothole is likely formed from a new, relatively more primitive pulse of magma that replenished the chamber. Data for normalization in (**a**) are from ref. ^66^. Abbreviations: Opx, orthopyroxene; Pl, plagioclase; Cpx, clinopyroxene; Ap, apatite; Mgt, magnetite; Ilm, ilmenite; Qtz, quartz; Chr, chromite. Original data are reported in the electronic Tables S1–S3.

Potholed and footwall anorthosite layers consist of up to 92–96 vol. % plagioclase crystals (Figs. 2a,b and 3a) with texturally equilibrated boundaries (Fig. 2c) and the local development of a mottled texture produced by large oikocrysts of ortho- and clinopyroxene (Fig. 2a,b). Element mapping of anorthosite from the pothole shows little chemical zonation in plagioclase crystals (Fig. 2d). Anorthosites also reveal chondrite-normalized REE patterns with a prominent positive Eu anomaly that are similar in terms of the overall shape and abundance to those of their plagioclase crystals (Fig. 3b). In addition, the average Mg-numbers of oikocrysts of clino- and orthopyroxene in anorthosites are either similar (79–87%) to or only slightly more evolved (67–81%) than cumulus orthopyroxene in adjacent norite/orthopyroxenites (77–88%) (Fig. 3c). These features indicate that anorthosites are almost perfect adcumulates to heteradcumulates with very little material crystallized from a trapped liquid. In this respect, these anorthosites are very similar to those of the Stillwater Complex, which are also extremely depleted in all incompatible elements due to very low volumes (a few percent) of a crystallized trapped liquid^22^. Another important feature is that anorthosite/leuconorite in the pothole as well as orthopyroxenite in direct contact with it are distinguished by much higher average An-content of plagioclase (80–85%) compared to that in footwall anorthosite/leuconorite (73–78%) (Fig. 3b,c).

## Generation of Plagioclase-Only-Saturated Melts by Ascent-Induced Decompression

We suggest that the observed transgressive relationships (Fig. 1) can be best explained as a result of the replenishment of the crystallizing Bushveld chamber by basal flows of highly reactive and, most likely, superheated (with no crystals) melts that caused physical disruption and subsequent thermo-chemical erosion of the cumulates on the chamber floor. The high erosive power of the replenishing melts can be attributed to their chemical and thermal disequilibrium with the footwall rocks^23^. The marked reversal towards higher An-content of plagioclase (up to 85%; Fig. 3c) indicates that the melts must have had a distinct chemical composition compared to the resident melt in the chamber that crystallized the footwall rocks (orthopyroxenite/leuconorite/anorthosite). The erosion event was followed, upon cooling, by crystallization of anorthosites from the plagioclase-only-saturated melts. Our working hypothesis thus postulates the existence in nature of highly reactive melts, which upon cooling at low pressure, become saturated in plagioclase alone. The challenge is to explain the source of these melts and why they have plagioclase as the sole liquidus phase.

**Figure 4:**
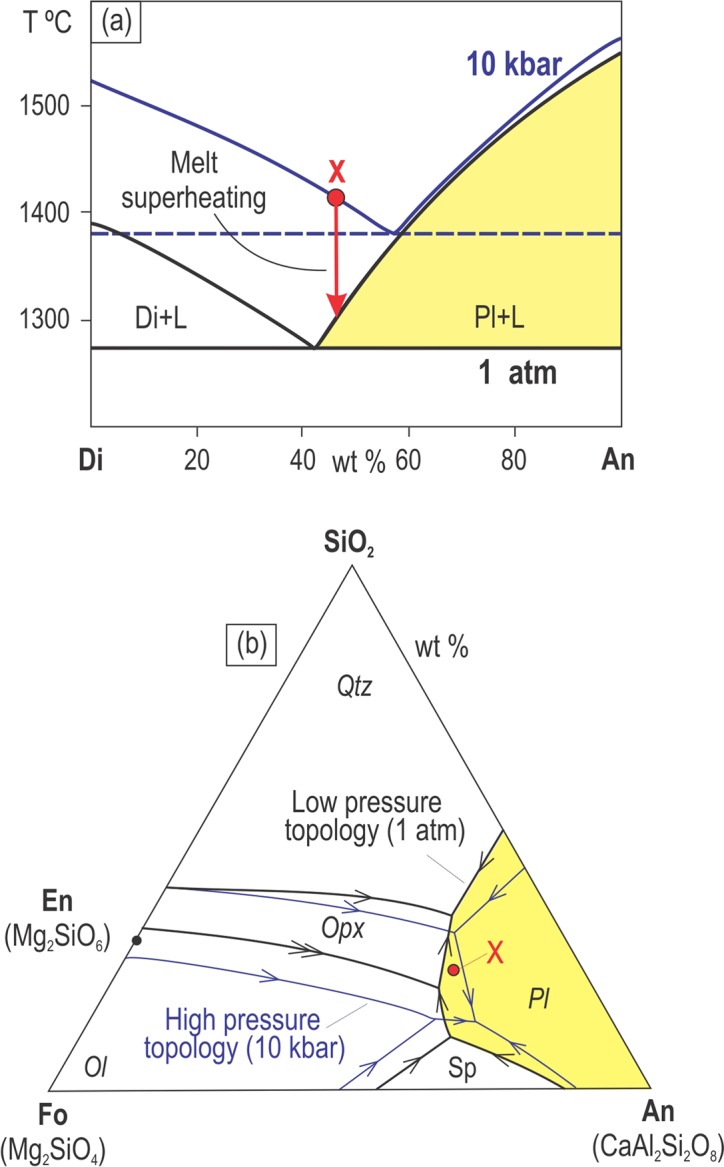
Liquidus phase diagrams illustrating the mechanism via which dry basaltic melts become saturated in plagioclase only by pressure reduction. (**a)** Effect of lithostatic pressure on the liquidus and eutectic compositions in the binary anorthite-diopside (An-Di) system at 1 atm and 10 kbar. The significant expansion of the stability field of plagioclase at the expense of clinopyroxene occurs with a decrease in total pressure. The diagram suggests that some liquids in equilibrium with clinopyroxene at high pressure (e.g. X) will precipitate only plagioclase at lower pressure. Also note that the decompressed liquid will be superheated relative to its liquidus temperature by more than 100 °C. Figure is compiled from ref. ^18^. **(b)** Effect of lithostatic pressure on the liquidus, eutectic and peritectic compositions in the ternary system forsterite-anorthosite-silica (Fo-An-SiO_2_) at 1 atm and 10 kbars. There is a significant expansion of the stability fields of plagioclase and olivine at the expense of orthopyroxene with a decrease in total pressure. The diagram suggests that some liquids in equilibrium with orthopyroxene at high pressure (e.g. X) will precipitate only plagioclase at lower pressure. Hereafter, cotectic and reaction lines are indicated by one and two arrows, respectively. Figure is modified from ref. ^25^.

It has been long understood from experimental data on simple binary (Fig. 4a) and ternary (Fig. 4b) systems that a decrease in lithostatic pressure may force dry liquids from the stability fields of mafic phases (e.g. pyroxene) into that of plagioclase alone^24,25^. This is due to expansion of the stability volume of plagioclase at the expense of mafic phases with decreasing pressure^24^. Importantly, the dry liquids in this process become superheated with respect to their liquidus temperature so that no crystals form (Fig. 4a). A similar expansion of the stability volume of plagioclase is also expected for water-saturated melts subjected to a drop in water pressure. However, this process results in an abrupt increase in liquidus temperature of these melts causing strong constitutional supercooling and rapid polybaric crystallization^26,27^. In contrast to dry melts, a decompression-induced degassing may result in extensive crystallization of plagioclase in water-saturated melts^26,27^ which could perhaps explain the formation of anorthosite layers. In volcanic arcs, this process also results in the formation of degassed magmas with a high content of intratelluric phenocrysts of plagioclase and other minerals (i.e. crystal-rich slurries). This scenario is not, however, applicable to our case. The parental magmas of the Bushveld Complex are considered to be relatively dry because of the paucity of liquidus phases rich in water, such as amphibole or biotite^19^. In addition, crystal-rich slurries would not be able to cause thermo-chemical erosion of pre-existing cumulates in magma chambers because phenocrysts would rapidly settle to the floor and protect the footwall from erosion^21^. Finally, the deposition of plagioclase phenocrysts from crystal-rich slurries on the chamber floor would result in the rapid accumulation of a thick mushy layer – a physical situation that would prevent the adcumulus growth of monomineralic anorthosites (see below). We believe therefore that the ‘dry’ melt scenario is a much more likely option for our case.

Although well-known, the expansion of the stability volume of plagioclase with reduction in lithostatic pressure – quite astonishingly – has never been invoked to explain the formation of melts parental to stratiform anorthosites in layered intrusions. This is likely because of the difficulty in envisaging a plausible mechanism for a substantial reduction in pressure *within* a magma chamber^14,28^: the pressure drop is generally expected to be small because the crust is weak and cannot sustain large overpressure. The overpressure in a magma chamber - i.e. the magma pressure in excess of the lithostatic pressure at the time of rupture - is normally equal to the tensile strength of the host rock of the reservoir and is commonly only about 3 MPa^29^. There is, however, a simple option that has been hitherto overlooked: it is not the resident melts in the shallow-level chambers that undergo a pressure reduction, but rather the new melts ascending from depth, i.e. deep-seated magma chambers. We propose that plagioclase-only-saturated melts parental to stratiform anorthosites are not produced endogenously within the shallow-level chambers but rather are generated within, or on the way up from, deep-seated sites of magma storage. The idea about external derivation of melts parental to stratiform anorthosites is not entirely new: it was first put forward by Irvine and his co-workers about forty years ago^30,31^ although it is still not recognized by igneous petrologists as a feasible idea. We believe, however, that new observations warrant reconsidering this common notion. A first step in this direction is to examine thermodynamically if dry melts parental to cratonic layered intrusions and ascending towards the Earth’s surface may become first superheated and then saturated in plagioclase only upon cooling in the shallow-level chambers.

**Figure 5:**
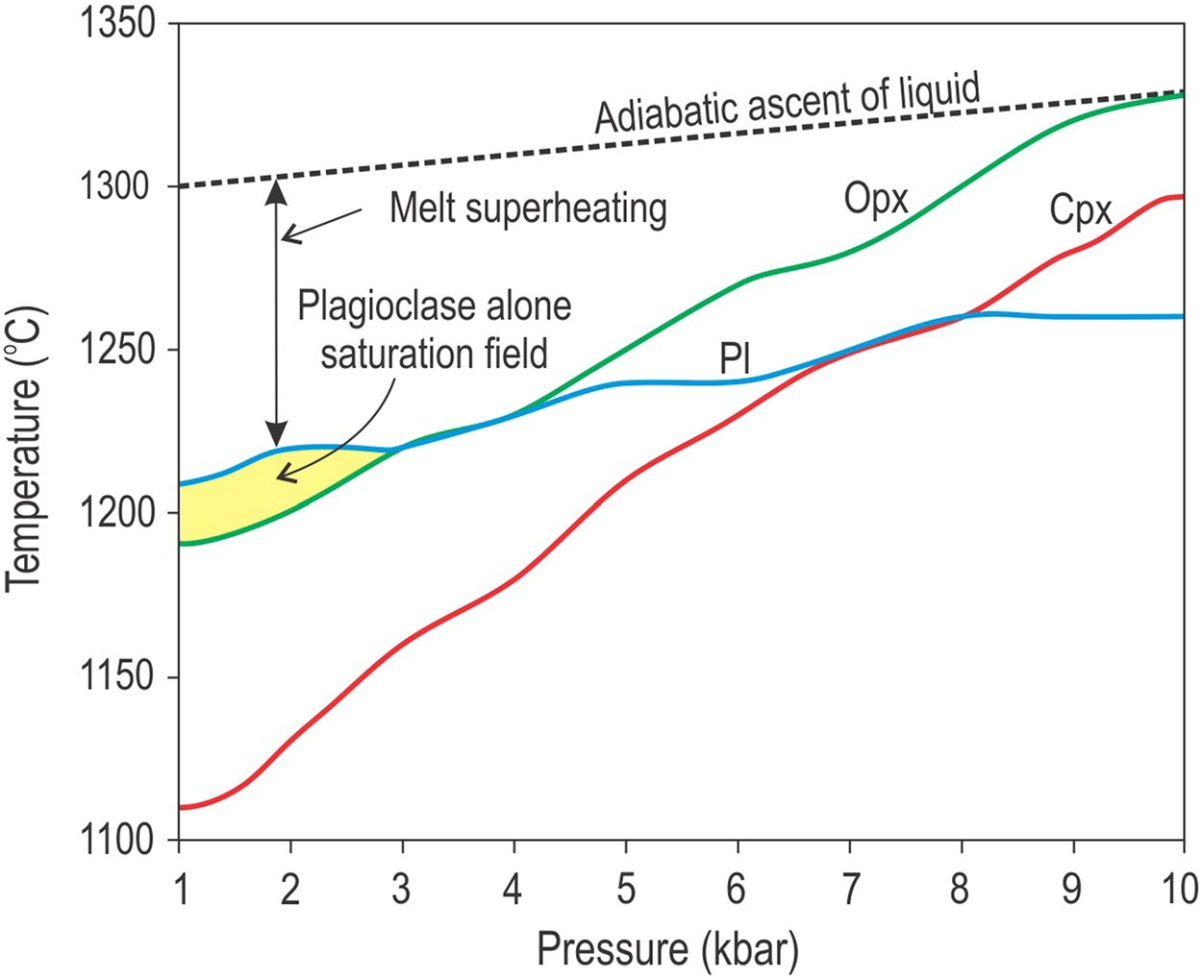
Thermodynamic modelling illustrating the mechanism via which alumina-rich, basaltic andesitic melts become saturated in plagioclase only by pressure reduction. The curves show the temperature of the first appearance for each mineral phase at any pressure from 1 to 10 kbar. The alumina-rich basaltic andesitic melt saturated in orthopyroxene at high pressure conditions (along the interval from 10 to 3 kbar) becomes saturated in plagioclase alone as the first liquidus phase, followed by orthopyroxene, at low pressures (P < 3 kbar). Note that adiabatic ascent of the crystal-free melts from a deep-seated storage region towards the Earth’s surface may result in up to 90 °C of melt superheating with respect to their liquidus temperature. Crystallization of the melt (Table 1) and adiabatic ascent was modelled using the MELTS algorithm under FMQ oxygen buffer conditions and a low water content (0.55 wt.%) using alphaMELTS software, version 1.4.1 (Supplementary Materials). The results of modelling are summarized in the electronic Table S5.

To explore this scenario, we have undertaken thermodynamic modelling using the alphaMELTS software (Supplementary Materials), with a focus on anorthosites from the UCZ (Figs. 1–3). The rock sequence in the UCZ is inferred to have parental melts that are orthopyroxenitic to noritic in composition^19,32^ and which are likely produced by contamination by alumina/silica-rich crustal rocks and fractional crystallization of primary basaltic or komatiitic magmas in deep-seated staging chambers^33,34^ (Supplementary Materials). Starting from one of these compositions (B2 melt^32^) we incrementally and iteratively modified its major element contents (Methods) to explore whether isobaric crystallisation at different pressures (from 10 to 1 kbar) of the obtained melts may identify a pressure interval within which plagioclase is the sole liquidus phase. One of these liquids is listed in Table 1. According to the TAS diagram, this liquid should be classified as a basaltic andesitic melt (SiO_2_ = 54.17 wt.%; Na_2_O + K_2_O = 2.64 wt.%) enriched in alumina (Al_2_O_3_ = 20 wt.%); it is saturated in orthopyroxene along the pressure interval from 10 to 3 kbar (Fig. 5) but has plagioclase as the first liquidus phase, followed by orthopyroxene, at pressure lower than 3 kbar. At 2 kbar, the temperature interval between the first appearance of liquidus plagioclase and orthopyroxene is 20 °C which corresponds to fractional crystallization of 4 wt.% of pure plagioclase. A 100 m thick column of such melt would thus produce an ~4 m thick layer of anorthosite. The formation of a 10 m thick anorthosite layer, as shown in Fig. 1a, would require a 250 m thick column of the plagioclase-only-saturated melt. Plagioclase (An = 81%) and orthopyroxene (Mg-number = 85.5) that crystallize from this melt at 2 kbar are compositionally close to those observed in the rocks of the UCZ^19^ (Fig. 3). In this process, the melt is expected to become superheated by up to 90 °C if it ascends from the deep-seated reservoir (~10 kbar) nearly adiabatically (Fig. 5). Thus, the results of our modelling entirely corroborate the key inference from experimental data on simple binary/ternary systems: alumina-rich basaltic andesitic melts located in the orthopyroxene stability field at high pressure become initially superheated and then, upon cooling, saturated in plagioclase alone at low pressure (Fig. 5). We also infer that the magmas parental to a large spectrum of plagioclase- and orthopyroxene-rich rocks (anorthosite, norite, leuconorite, melanorite and orthopyroxenite) of the UCZ of the Bushveld Complex were likely formed from compositions ranging from alumina-rich basaltic to basaltic andesitic melts (Supplementary Materials).

**Table 1:** Composition of alumina-rich, basaltic andesitic melt (a new proposed parental composition B2* for the UCZ) used for alphaMELTS modelling at a pressure range from 1 to 10 kbar.

Composition	SiO_2_	TiO_2_	Al_2_O_3_	Fe_2_O_3_	FeO	MgO	CaO	Na_2_O	K_2_O	H_2_O	Total
Basaltic andesitic melt	54.17	0.13	20.00	0.63	4.89	7.00	10.00	2.50	0.14	0.55	100.00

## A Novel Hypothesis for Origin of Stratiform Anorthosites in Layered Intrusions

### Parental melts to stratiform anorthosites in the Bushveld Complex

**Figure 6:**
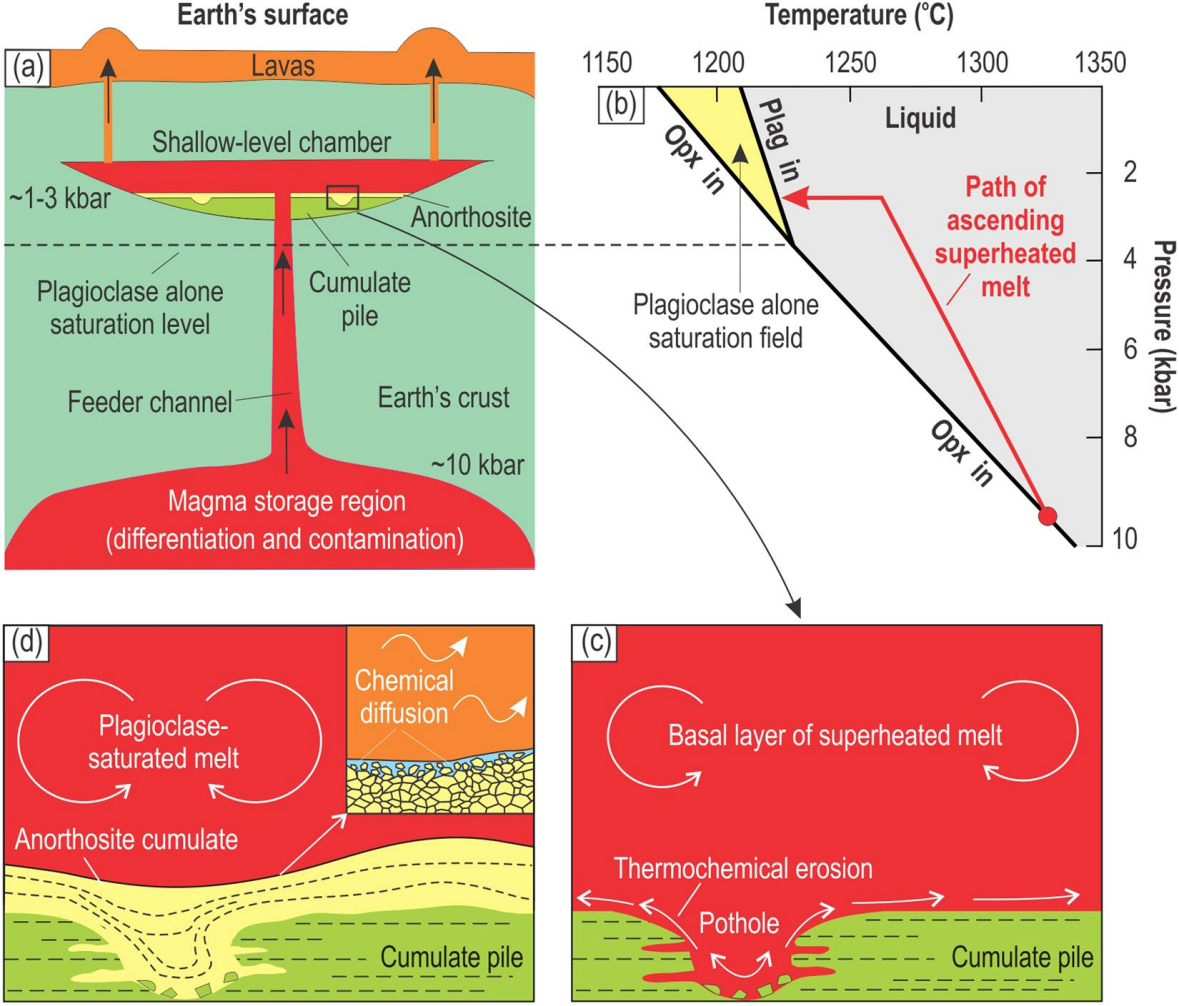
A physical model for the formation of stratiform anorthosites from basaltic andesitic melts saturated in plagioclase only due to reduction in lithostatic pressure. (**a)** Basaltic andesitic melts ascending from lower crustal storage regions experience reduction in lithostatic pressure. This results in expansion of plagioclase stability volume and, as a result, some basaltic andesitic melts saturated in orthopyroxene at high-pressure regions may become, after some cooling, saturated in plagioclase alone at lower pressure in shallow chambers. Early and almost perfect fractional crystallization of these plagioclase-saturated melts, in an open system where magma can also flow out of the chamber, will produce monomineralic layers of anorthosite. **(b)** Phase relations for a basaltic melt in P-T space illustrating the model that basaltic andesitic melts saturated in orthopyroxene first become slightly superheated during their ascent and then saturated in plagioclase alone after stalling and cooling in shallow-level chambers. Therefore, allowing for the development of stratiform anorthosite in shallow-level chambers. The phase diagram is simplified from Fig. 5 and is used to graphically illustrate the principle lying at the heart of our model. **(c)** A dense and superheated melt entered the chamber and spread across the floor of the chamber as a basal layer causing local rupturing of the chamber floor and formation of pull-apart structures. The melt caused thermochemical erosion of cumulates at the temporary floor of the chamber, widening pull-apart structures into sub-rounded potholes. **(d)** On cooling, the melt crystallized plagioclase *in situ*, i.e. directly on the irregular erosional surface, including potholes. Near perfect anorthosite adcumulates form owing to exchange between thin boundary mushy layer and overlying melt via chemical diffusion aided by convection in the chamber.

Our novel hypothesis implies that parental magmas for the anorthosites in the Bushveld Complex are alumina-rich basaltic to basaltic andesitic melts which were derived as residual melts from deep-seated staging chambers (Supplementary Materials). These melts are produced there by fractional crystallization and crustal contamination of primary basaltic to komatiitic magmas. Both these processes tend to increase SiO_2_, Al_2_O_3_, CaO and Na_2_O and decrease MgO in the residual melts. The residual melts are considered to be saturated in orthopyroxene at high pressure in deep-seated staging chambers but become saturated in plagioclase when transferred into and cooled within a shallow-level Bushveld magma chamber at lower pressure (Fig. 6a). It is not possible to define the exact composition of these melts from the solid products of their crystallization (i.e. anorthosite cumulates), but one can define a range of potential melts that may behave in the way predicted by our hypothesis. For illustration purposes, we have used above a basaltic andesitic melt with 20 wt.% Al_2_O_3_ (Table 1) although the compositions with the lower alumina content (down to 15–16 wt.% Al_2_O_3_) show a similar behaviour. In general, these melts can be regarded as analogues to the so-called A_0_ melt (~18 wt.% Al_2_O_3_)^30^ – the high alumina basalt that was postulated as parental to most of the anorthosite and two-pyroxene gabbro in the Critical and Main Zones of the Bushveld Complex^31,35^. The composition of this A_0_ melt has been estimated from samples of fine-grained two-pyroxene gabbro in marginal facies of the Critical Zone ranging in composition from ~14 to ~19 wt.% Al_2_O_3_^35^. The gabbro consists of plagioclase, clinopyroxene, and orthopyroxene, with many of the plagioclase crystals being arranged in weak radial growths indicating their quench crystallization^31^. Importantly, a low-pressure experimental study of phase relations in one of these gabbro compositions (~15.5 wt.% Al_2_O_3_) showed plagioclase as the first liquidus phase^31^.

Our thermodynamic modelling using alphaMELTS shows that the capacity of alumina-rich basaltic/basaltic andesitic melts to crystallize plagioclase as a first liquidus phase is sensitive to their chemical composition. In addition, even the melts with the ‘correct’ composition have a rather small stability field of plagioclase alone crystallization (Fig. 5, yellow area). All this suggests that this type of plagioclase-saturated melts is unlikely to be prevalent among those arriving from a deep-seated magma chamber. The prediction is consistent with the observation that anorthosites in the Bushveld Complex and other layered intrusions are much less common compared to other cumulates (e.g. norite, gabbronorite, orthopyroxenite). It seems therefore that only some of the ascending melts may reach plagioclase-only saturation: those richer in MgO compared to our initial melt composition (Table 1) will still remain saturated in orthopyroxene. These orthopyroxene-only-saturated melts can be parental to orthopyroxenites that are so common in the UCZ of the Bushveld Complex. To explain the sequences with alternating orthopyroxenites and anorthosites in the UCZ, one can envisage a scenario in which the evolution of residual melt in the staging chamber by fractional crystallization and crustal assimilation (making it more evolved) was complicated by rejuvenating influxes of mantle-derived magmas (making it more primitive). In this case, the residual melt escaping from the staging chamber at different stages of its back-and-forward changes in composition, may show slightly varying SiO_2_, Al_2_O_3_ and MgO contents. These small differences in the melt composition can drive the crystallization of either plagioclase or orthopyroxene as a first liquidus phase producing alternating stratiform layers of orthopyroxenites and anorthosites in the Bushveld chamber.

### A deep-seated staging magma chamber to the Bushveld Complex

Our concept implies the existence of a large underlying chamber that supplies the residual melts into the overlying Bushveld chamber. It also requires the continental crust to be thick enough to ensure a large distance between the deep-seated and shallow chambers so that the residual melts would experience a substantial pressure drop when travelling from one chamber to another. The deep-seated staging chamber beneath the Bushveld Complex has long been postulated by petrological models^34,36–38^ but has only recently been imaged using seismic and gravity data^39^. The remnants of the staging chamber of ~10 km in thickness were identified at a depth of ~40–45 km, with its base coinciding with the Moho discontinuity^39^. Considering that the Bushveld chamber was emplaced at a depth of ~5 km, it leaves ~25–30 km for the melts to travel from the deep-seated staging chamber towards the shallow Bushveld chamber. This depth difference translates into a pressure drop of ~7.5–9.0 kbar (3.3 km = 1 kbar) which is in the range of a pressure decrease predicted by our model (Fig. 5). It should be noted in passing that in our model a decompression can be anywhere from 10 to 2 kbar. For instance, a melt from a pressure region of about 5 kbar having the bulk composition (liquid and crystals) as in Fig. 5 will reach the field of plagioclase alone saturation by a pressure drop of only 3 kbar.

### The basal magma emplacement and thermochemical erosion of floor cumulates

The new melts from the deep-seated staging chamber are envisioned to enter the Bushveld chamber as dense basal flows (up to a few hundreds meter thick) along the chamber floor with little to no mixing with the overlying resident melt saturated in orthopyroxene. The plagioclase-saturated melts are considered to be denser than the resident orthopyroxene-saturated ones because the latter are normally richer in normative quartz^30^. In addition, the resident melt is expected to be lighter because of being richer in water, which dramatically decreases the density of silicate melts^40^. The basal emplacement of new melts is fully consistent with intensive erosion of floor cumulates which is only possible if the inflowing melts come in direct contact with the rocks of the chamber floor^21,41^. Some mixing of new melts with the resident melt will likely still occur upon entry into the chamber and this may increase the initial superheating of the replenishing melt^30,42,43^. We believe, however, that the magma mixing is unlikely to be significant because otherwise it would drive the composition of the hybrid melt outside the plagioclase alone stability field making the formation of anorthosites impossible. Alternatively, one can argue that only a small portion of the melts which were fortunate to escape the mixing with the resident melt were able to crystallize the stratiform anorthosites.

The basal flows of new replenishing melts are in thermal and, most importantly, chemical disequilibrium with the floor cumulates in a shallow-level chamber and are therefore highly reactive. Thermal disequilibrium is due to melt superheating that can be up to 90 °C for the melt rising from the deep-seated reservoir near adiabatically (Fig. 5). In reality, some cooling of rising melts will inevitably occur so that upon arrival into a shallow-level chamber they may be much less superheated (Fig. 6b). Simple calculations illustrate, however, that even at a low degree of superheating (say, 5–10 °C) a few hundreds meters’ thick melt column is capable of eroding thermochemically a couple of dozens of meters of pre-existing footwall rocks^21,41,44^. This is because a major agent of the erosion is not the superheat itself but rather chemical disequilibrium between the new melts and floor cumulates: the predominant erosional process is dissolution rather than melting of the floor. Melt superheating is still important because the non-superheated replenishing melts are expected to start crystallizing soon after their arrival into the chamber and will therefore form a new layer of rocks to cover the pre-existing floor cumulates and shut down the erosion of the floor cumulates. For this reason, the field relationships (Fig. 1) require the replenishing melts to have been superheated, otherwise it would not have been possible to produce potholes by erosion^21,41,44^.

There is yet another important aspect of the erosion process to consider. The erosion can be effective only if the melt released by dissolution of footwall rocks convects away from the surface where their melting is taking place. However, the melt produced by dissolution of orthopyroxenite is expected to be denser than the overlying melt. There is thus a danger that this dense melt will pond at the floor of the chamber and terminate further erosion of orthopyroxenites. One way in which dissolution may still operate effectively is if the floor is inclined and the melt released by dissolution flows away downslope. Some petrological studies^45^ do provide evidence for a sloping temporary floor to the chamber of several degrees at the time of the formation of the Critical Zone, thus eliminating the physical obstacle for thermal/chemical erosion of the pyroxenitic footwall rocks.

The transgressive relationships between anorthosites and their footwall cumulates in the studied pothole (Fig. 1) can thus be attributed to thermochemical erosion at the initial point of weakness (such as a pull-apart structure) likely produced by rupturing of the floor cumulates due to the load of replenishing magma^45^ (Fig. 6c). Note, however, that the erosion and contamination of the melt by footwall cumulates is not considered here as a trigger for saturation of the melt in plagioclase. Just like magma mixing, the substantial contamination of a new melt by footwall orthopyroxenite will tend to decrease its capacity to crystallize anorthosite due to driving its composition outside the plagioclase-alone stability field. A simple way to avoid this is to suggest that the melt that crystallized monomineralic anorthosite is not the same one that previously eroded the footwall. It is quite conceivable that the initial melt that caused the erosion may have been flushed away by new batches of melt entering the chamber. Yet, another possibility to avoid assimilation (not related to the studied case), is to suggest that some new melts may lose most of their superheat during the transcrustal ascent and will therefore start crystallizing anorthosites immediately after their arrival into the chamber. Some anorthosite layers with no obvious erosional relationships^19,28^ with their footwall rocks can be probably attributed to the formation from such non-superheated melts.

### Formation of monomineralic anorthosite at the chamber floor by adcumulus growth

After some cooling, the melt started finally crystallizing plagioclase either directly along the eroded surfaces and/or within a basal layer with subsequent deposition of crystals on the chamber floor (Fig. 6d). The accumulation of plagioclase crystals on the chamber floor is not yet sufficient, however, to produce monomineralic anorthosites. It is also necessary to get rid of nearly all evolved interstitial liquid between plagioclase crystals. The interstitial liquid (or rejected solute) produced by plagioclase crystallization is expected to be denser than the original melt and therefore intrinsically stagnant on the chamber floor. Consequently, there is no way to remove the interstitial liquid from plagioclase crystals by compositional convection to produce anorthosite adcumulates if the floor is completely flat. As mentioned above, there are, however, data indicating that the temporary floor at the time of the Critical Zone formation was sloping at several degrees^45^ which may allow the interstitial melt released by crystallization to flow away downslope. It should be noted, however, that adcumulus growth can be effective even at the flat floor situation. Morse^46^ showed that in large magma chambers the characteristic transport distance for chemical diffusion alone (3–4 cm/year) is up to eight times higher than the rate of accumulation at the chamber floor (0.5–1.0 cm/year). It is therefore quite probable that anorthosite adcumulates with very low residual porosity can form directly at the crystal-liquid interface of the flat chamber floor by diffusion alone^46^ (Fig. 6d, insert). In addition, the high Mg-number of pyroxene oikocrysts indicates that these are not truly intercumulus phases produced from a trapped liquid within anorthosites. Rather, these are heteradcumulate minerals, i.e. cumulus phases that have grown in a thin mushy layer in direct contact with the overlying body of flowing or convecting melt^47,48^. Heteradcumulate minerals may only form in this way when interstitial melt can freely communicate with the overlying resident melt. In contrast, the phases that crystallize deep in the thick mushy pile will have a quite limited, if any, interaction with the overlying resident melt and therefore their final compositions will be quite evolved. This is, again, indicative of the formation of adcumulate anorthosite at a crystal-liquid interface under conditions of a very low rate of crystal accumulation^46,48^. The melt remaining after the formation of monomineralic anorthosites can be either mixed with a new replenishing melt that terminates the crystallization of anorthosites or, alternatively, flushed away to erupt as lavas via volcanos (now eroded away) above the Bushveld Complex (Fig. 6a). Both scenarios are in line with a current consensus among igneous petrologists that most layered intrusions are growing incrementally via numerous magma pulses that pass through the evolving magmatic chambers^8^.

### Comparison with other existing models for the formation of stratiform anorthosites

Our novel proposal is advantageous over the earlier attempts to address the problem of the generation of plagioclase-only-saturated melts^14,49,50^ in that it relies on a physical process that happens in nature almost inevitably. All mantle-derived melts, including those residing and contaminated in the deep-seated staging chambers, ascend towards the Earth’s surface and none can avoid reduction in lithostatic pressure during this process. Therefore, it is quite natural that some ascending alumina-rich basaltic andesitic melts may reach plagioclase-only saturation upon their ascent and subsequent cooling. In some respects, our concept can be regarded as the development of the earlier model by Irvine and his co-workers^30,31^ who attributed the stratiform anorthosites in the Bushveld and Stillwater Complexes to periodical replenishment of the evolving chamber by A-type (‘A’ - anorthositic) magma which – for reasons unspecified by those authors – was also initially saturated in plagioclase only. This model implies that the new A-type magma was geochemically and even isotopically distinct from the resident U-type magma (‘U’- ultramafic) that crystallized mafic-ultramafic rocks in the chamber. This elegant concept was, however, rejected by the subsequent detailed geochemical studies of the Bushveld Complex. It turned out that anorthosites and associated norite, orthopyroxenite and dunite/harzburgite are geochemically and isotopically consanguineous rocks^51,52^ suggesting their formation from the same parental magma, rather than from two magmas of different lineages. This inference was further supported by similar composition of plagioclase (e.g. An-content) and pyroxenes (e.g. Mg# and Cr/Al ratio) in all these cumulate rocks^38^. Our model allows anorthosites to be cumulates of the externally-derived and plagioclase-only-saturated melts that have been replenishing the chamber^30,31^. We alleviate a major problem of the earlier proposal^30,31^ by suggesting that both anorthosites and associated mafic rocks in the UCZ of the Bushveld Complex are produced from derivatives of the same parental magma(s). These magma(s) were derived from the deep-seated staging chamber (Fig. 6) and crystallized cumulate rocks with different mineral assemblages (e.g. anorthosite, orthopyroxenite, norite) due to some variations in chemical composition.

Our new concept can be considered as a viable alternative to a current tendency to attribute stratiform anorthosites in layered intrusions to a mechanical segregation of plagioclase crystals from co-existing mafic phases within internally- or externally-derived crystal-rich slurries^20,36,53,54^. In these slurry models, the transgressive relationships of anorthosites with underlying rocks in the Bushveld Complex (Fig. 1) are attributed to sill-like emplacement of plagioclase-rich slurries (with ~50% plagioclase) into solidified footwall cumulates^20,55^. The slurries are derived either by large-scale slumping of the cumulate pile induced by chamber subsidence^20^ or partial melting of noritic rocks by syn-magmatic ultramafic sills^55^. The near monomineralic composition of anorthosites are explained by rapid and efficient draining of most interstitial liquid from the plagioclase-rich mush into footwall rocks prior to solidification^20^. However, it has been recently shown both geologically^21,41,44^ and texturally^56^ that a mushy zone at the top of the cumulate pile in the Bushveld Complex is almost non-existent (<4 m thick)^56^, leaving no possibility for large-scale slumping processes in a cumulate pile and for the removal of the liquid component from the postulated slurry^20,54^. Similarly, both field and chemical observations^57,58^ are not supportive of the idea that the ultramafic units of the Bushveld Complex are synmagmatic sills depriving this model of a heat source to re-melt noritic rocks to produce a plagioclase-rich mush^55^.

## Concluding Remarks

The transgressive relationships of stratiform anorthosites with their footwall rocks in the UCZ of the Bushveld Complex are best explained by the replenishment of the chamber by relatively dry, alumina-rich basaltic/ basaltic andesitic melts that were derived from deep-seated magma reservoirs. Some of these melts became superheated in response to pressure reduction during their ascent. Upon emplacement into the chamber as basal flows, these melts caused thermo-chemical erosion of pre-existing footwall rocks in the chamber. The erosion was mostly due to chemical disequilibrium between the new melts and floor cumulates, with the superheat assisting in keeping the melt away from the onset of its crystallization. On cooling, the melts reached saturation in plagioclase only and its subsequent adcumulus growth at the chamber floor resulted in the formation of monomineralic anorthosite. The process that is likely responsible for the melt saturation in plagioclase is thought to be the expansion of the plagioclase stability volume due to lithostatic pressure reduction. The expansion takes place when these melts – after some differentiation and crustal contamination in deep-seated magma reservoirs – are transferred upwards into the shallow-level Bushveld chamber. We propose that many stratiform layers of monomineralic anorthosites in mafic-ultramafic layered intrusions may be associated with a lithostatic pressure-induced shift of multi-saturated melts into the stability volume of plagioclase alone during their journey through the Earth’s crust. It should be emphasized that our model primarily applies to monomineralic anorthosites that occur as thick and continuous stratiform anorthosites in mafic-ultramafic intrusions and may not be extended to non-stratiform cases of monomineralic cumulates, especially in other types of intrusive complexes (e.g. massif-type anorthosites^59^). For instance, a few cm thick rims of monomineralic anorthosite that are locally developed below the Merensky Reef and massive chromitites in the Bushveld Complex are better explained by re-melting/selective dissolution of footwall noritic cumulates^21,41^. Prominent examples of discordant bodies of monomineralic anorthosite in the Skaergaard intrusion^60^ or Stillwater Complex^61^ are most likely produced by metasomatic replacement of pre-existing cumulates by late migrating fluids^60,61^. In a similar manner, fine-scale mineral-graded layering involving anorthosites in both layered intrusions^9,62–64^ and mid-ocean ridge crust^65^ require some alternative mechanisms, perhaps, involving oscillatory crystallization at crystal-liquid interface^62,65^ or crystal sorting in density magmatic currents^63^.

## Methods

### Rock sampling

Documentation of field observations and sampling of the anorthosite pothole at Smokey Hills mine, Eastern Bushveld was undertaken in November 2016. Seven samples (each sample weighed ~1–2 kg) were collected for petrographic, whole-rock (XRF and ICP-MS) and mineral compositional analyses (EMPA and LA-ICP-MS). Location of samples relative to the pothole structure is shown in Fig. 1a. Three samples come from the pothole (Bu-5/5 and 5/7 – anorthosites; Bu-5/6 – leuconorite) and one sample is taken directly at the base of the pothole (Bu-5.4 – orthopyroxenite). Three other samples are collected from footwall rocks (Bu-5/1 and 5/2 – anorthosites; Bu-5/3 – leuconorite). Our strategy for the sampling was to test if whole-rock and mineral compositions in the anorthosite/leuconorite forming the pothole are indicative of their formation from a new magma pulse replenishing the chamber.

### XRF imaging of rock samples

Microbeam scanning X-ray fluorescence analysis was conducted on a polished rock slab using the Bruker Tornado desktop scanner equipped with a rhodium target X-ray tube operating at 50 kV and 500 nA without filters and an XFlash silicon drift X-ray detector at CSIRO, Perth in Australia^44,48^. Analyses were quantified using the Bruker ESPRIT software, which provides standardless semi-quantitative analyses for elements heavier than Na, with estimated detection limits for the given operating conditions and dwell times of approximately 1000–2000 ppm for first-row transition metals. Maps were created using a 25–40 μm spot size on a 40 μm raster with dwell times of 10–20 ms per pixel. Maps are represented as un-quantified background-corrected peak height data for Kα peaks for each element.

### XRF and ICP-MS analyses of major and trace elements

. Major and trace elements were analysed by Intertek Genalysis in Australia. Major elements were determined by X-ray fluorescence analyses (XRF) using fusion disks (glass beads) fused with a lithium borate flux. This method is superior to pressed powders as it eliminates particle size and mineralogical effects which compromise the accuracy of power analyses. XRF calibrations were performed using high purity reference materials to which the calibration and analytical data are traceable. Corrections were made for drift, background, emission line overlap and inter element enhancement-absorption effects. Analytical process control was achieved using certified reference materials which are grade and matrix matched as far as possible. Calibrations used the international rock standards SARM8, SARM4, OREAS 24b, GBAP-12 as well as in-house controls. Agreement with recommended values was better than 0.6% for Cr_2_O_3_, Fe_2_O_3_, MgO, Al_2_O_3_ and better than for 1–6% for all other major elements. Trace elements were determined using 4 acid digests of the material and inductively-coupled plasma mass spectrometry (ICPMS) and optical emission spectrometry (ICPOES). Calibrations were made using certified reference material solutions. Each ICP-MS analysis was accompanied by control standards GTS-2a, AMIS0167, and AMIS0013 and selected samples were re-analyzed to check anomalous results. Corrections for drift were made using internal standards. Corrections for interferences were accomplished using collision cell technology (ICPMS) and IEC models. Various certified reference materials are used in the analytical process control. Pulp replicates (checks) were selected during job registration to check the data repeatability. Selected samples are routinely re-analysed to confirm anomalous results. For all elements, the relative standard deviations were less than 10%. A complete list of analyses is available online within the electronic Table S1.

### Electron probe microanalysis of minerals

Minerals were measured with a Cameca SX100 electron probe microanalyzer (EPMA) at the Institute of Mineralogy, Leibniz University, Hannover. We used a focused beam (1 µm) with a current of 15 nA and an accelerating voltage of 15 kV. Counting time on peaks was 15–20 s (7.5–10 s for background on each side of the peak) for each element. The following standards were used for for Kα X-ray line calibration: albite for Al and Na, orthoclase for K, wollastonite for Si and Ca, TiO_2_ for Ti, Fe_2_O_3_ for Fe, MgO for Mg, Mn_3_O_4_ for Mn. Detection limit is ~1000 ppm. Errors are within 1% relative of international standards. Raw data were corrected with the CATZAF software. A complete list of analyses is available online within the electronic Table S2.

### Laser ablation inductively coupled plasma mass spectrometry

*In situ* trace element analyses of plagioclase and pyroxenes were performed by LA-ICP-MS at the Institute of Mineralogy, Leibniz University, Hannover^13^. We used a ThermoScientific ElementXR fast-scanning sector-field ICP-MS coupled to a laser ablation system based on a Spectra Physics Solstice 194 nm femstosecond (fs) laser. Samples were ablated by rastering a 30 µm laser spot over areas of 100×100 µm with a laser repetition rate of 20 Hz. Laser sampling was performed in a He-Ar atmosphere. The following isotopes were then measured using the ICP-MS in low mass resolution mode: ^23^Na, ^25^Mg, ^27^Al, ^29^Si, ^31^P, ^39^K, ^43^Ca, ^45^Sc, ^47^Ti, ^49^Ti, ^51^V, ^53^Cr, ^55^Mn, ^57^Fe, ^60^Ni, ^85^Rb, ^88^Sr, ^89^Y, ^91^Zr, ^137^Ba, ^139^La, ^140^Ce, ^141^Pr, ^143^Nd, ^147^Sm, ^153^Eu, ^157^Gd, ^159^Tb, ^163^Dy, ^165^Ho, ^167^Er, ^169^Tm, ^173^Yb and ^175^Lu. Five mass lines were each measured for 5 ms in the peak centre of each isotope, resulting in a total sweep time of ∼1 s. Oxide formation rates were monitored by measuring ThO/Th ratios, which were always 0.1–0.4%. Signals were acquired for a total of 120 s per analysis with the laser off for the first 40 s in order to determine background count rates. Note that samples were pre-rastered before analysis to remove surface contamination. A 120 s gas rinse-out time was used after pre-rastering to allow element signals to return to baseline levels. SiO_2_ contents determined by EPMA were used as an internal standard (^29^Si), and the BIR-1G glass standard was used for quantification. Measurements of the BCR-2G glass standard indicate that most elemental analyses were accurate to better than 10% relative, with only P, Cr and Cu returning deviations of >10% relative. A complete list of analyses is available online within the electronic Table S3.

## Supplementary information

Supplemental information 1.

Supplemental information 2.

Supplemental information 3.

Supplemental information 4.

Supplemental information 5.

Supplemental information 6.

## Data Availability

All data generated or analyzed during this study are included in this published article (and its Supplementary Materials Files).
