# Supplementary material for: Monomineralic anorthosites in layered intrusions are indicators of the magma chamber replenishment by plagioclase-only-saturated melts
Source: Sci Rep. 2020 Mar 2;10:3839. doi: 10.1038/s41598-020-60778-w (PMC7052229; doi:10.1038/s41598-020-60778-w)
Supplement: Supplementary file 1 — Supplemental information 1. [file 41598_2020_60778_MOESM1_ESM.pdf]

## SUPPLEMENTARY MATERIALS

### Parental melt composition and thermodynamic modelling using MELTS program

All thermodynamic calculations of crystallization sequences in this study were carried out using alphaMELTS software, version 1.4.1[67,68]. Our modelling aimed at identifying the melt composition that can be parental to the rocks of the UCZ of the Bushveld Complex. The melt should produce at low pressure a sequence of rocks mostly consisting of orthopyroxene and plagioclase (anorthosite, norite, leuconorite, melanorite and orthopyroxenite); also, both modal proportions and mineral composition should be similar to those observed in the UCZ cumulates. Keeping these constraints in mind, we have examined and rejected several basaltic compositions, which did not satisfy the above conditions. The result is not surprising, as a large number of experiments and/or thermodynamic calculations on melting mantle peridotite to generate primitive basalts clearly show that the first liquidus mineral at both low and high pressure, is olivine[1,18,69-81]. Since olivine is rare to nearly absent in the UCZ, it is clear that the parent liquid was not a primary mantle-derived basalt, and therefore we re-directed our search for the starting composition from primary mantle-derived basalts towards derivative liquids that have compositions close to high-alumina basaltic to basaltic andesitic melts.

The experimental data[81] show that high-alumina basalts have liquidus clinopyroxene followed by plagioclase at high pressure, while at low pressure plagioclase is a liquidus phase followed by olivine and then clinopyroxene. This supports a major claim of our study regarding the change in the first liquidus phase with pressure. However, these results[81] are not applicable for the UCZ of the Bushveld Complex in which olivine is almost absent and clinopyroxene is a minor late-stage phase. We drew therefore our attention towards earlier studies which considered marginal rocks of the UCZ as representing the chilled parental melts. One of these compositions, referred to as B2 melt, was earlier suggested as a possible parental melt for the UCZ[33]. By running the B2 melt composition (fig. S1, Table S4) we have found that at 10 kbar clinopyroxene (pigeonite) is the first liquidus phase, followed by plagioclase (but only after ~34 wt% clinopyroxene is already formed). At 2 kbar, the same B2 liquid produces spinel as a liquidus phase and then plagioclase at T = 1230ºC. After a short temperature interval clinopyroxene joins plagioclase and, at T ~1150ºC plagioclase can reach ~40 wt. %, together with ~30 wt% of clinopyroxene (fig. S2, Table S4). Again, the crystallization sequences of the B2 melt support our claim regarding the change in the first liquidus phase with pressure. However, ignoring the spinel, plagioclase is shortly followed by clinopyroxene and therefore the prevailing cumulate rock to form from this composition would be pigeonite gabbro to leucogabbro, rather than norite to orthopyroxenite as observed in the UCZ.

To produce rocks of the UCZ, the B2 composition needs to be modified in such a way that plagioclase and orthopyroxene would be the main phases to crystalize, with no olivine and little clinopyroxene. We have obtained such a liquid composition through backwards modelling (iterative trial and error runs with changing the concentration of some major elements) (Table 1). For this, both $SiO_2$ and $Al_2O_3$ in B2 melt should be increased by ~4 wt.% and FeO and CaO should be decreased by ~3 wt.% and ~1 wt.%, respectively. The thermodynamic calculations were run over a pressure range from 10 to 1 kbar at FMQ oxygen buffer and a low water content (0.55 wt.%). The temperatures of the first appearance of minerals for every run were compiled and plotted in a pressure-temperature diagram (Fig. 5). A complete data on thermodynamic modelling are presented in the Table S5. The results show that at low pressure (2 kbar), the modified B2 composition has plagioclase as the first liquidus phase shortly followed by orthopyroxene. Clinopyroxene starts to crystallize after a large amount of plagioclase and orthopyroxene is already formed. Under fractional crystallization conditions, these melts would produce anorthosite as the first-forming cumulate. Under equilibrium crystallization conditions, they would form norite to leuconorite, similar to those

observed in the UCZ. Based on these results, we propose that the parental melts for the UCZ of the Bushveld Complex were most likely of alumina-rich basaltic to basaltic andesitic composition (equivalent to noritic composition).

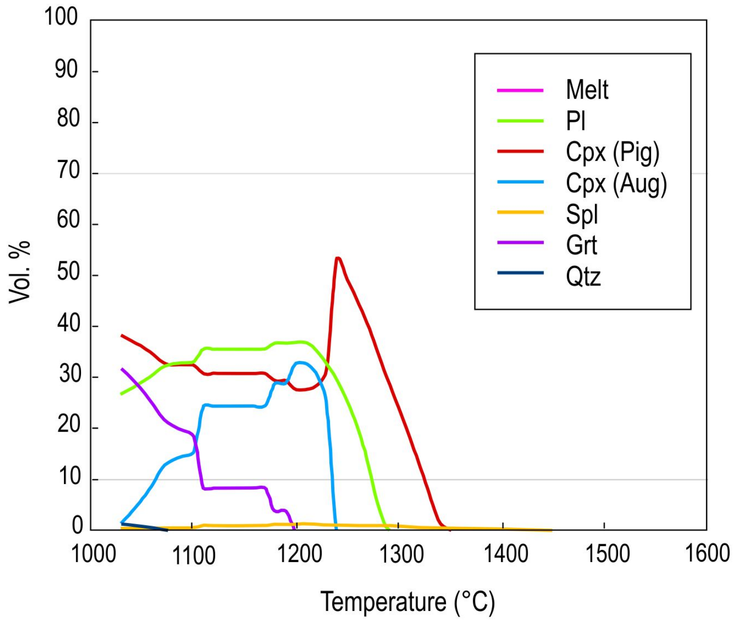


**Fig. S1.** Phase proportions for an isobaric run at 10 kbar of the B2 melt composition (*20*)

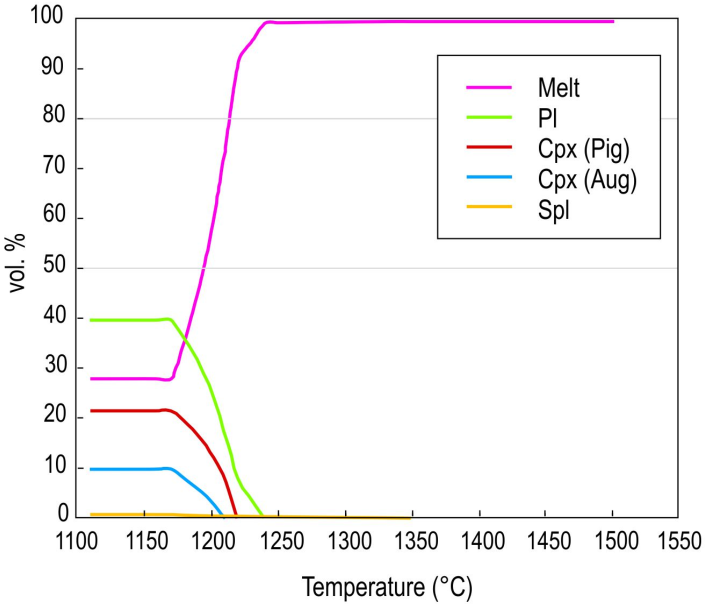


**Fig. S2.** Phase proportions for an isobaric run at 2 kbar of the B2 melt composition (*20*)

What remains to be clarified is the way in which such alumina-rich basaltic or basaltic andesitic melts can be generated, as it is clear from their high $SiO_2$, $Al_2O_3$, and low MgO (Table 1) that these cannot be produced by direct melting of mantle rocks, nor by fractional crystallization of normal primary basalts, alone. We suggest, therefore, that these melts are likely formed in a deep-seated chamber from mantle-derived basaltic or komatiitic[33,35,38] magmas which assimilated $SiO_2$- and $Al_2O_3$-rich crustal rocks prior to and/or during fractional crystallization of olivine and/or orthopyroxene. Various degree of assimilation together with fractional crystallization in the staging

chamber would control the composition of the liquids ascending towards the overlying Bushveld's chamber. In this way, the melts ranging in composition from alumina-rich basaltic to basaltic andesitic melts (similar to one in Table 1) can be generated. Such mixing-assimilation processes are probably quite common in the deep-seated staging chambers feeding the overlying mafic-ultramafic layered intrusions. The detailed consideration of the complex processes in deep-seated staging chambers is beyond the scope of this study but the general modelling approach is similar to that adopted in one of our earlier studies[35].

**References**


67. Smith, P. M. & Asimow, P. D. Adiabat_1ph: A new public front-end to the MELTS, pMELTS, and pHMELTS models. *Geochemical, Geophysical, and Geosystems* **6**, Q02004 (online) (2005).
68. Antoshechkina, P. M. & Asimow, P. D. AlphaMELTS 3.0 and the MAGMA website: educational and research tools for studying the petrology and geochemistry of plate margins, Abstract ED41B-0644 presented at 2010 Fall Meeting, AGU, San Francisco, Calif., 13-17 Dec. (2010).
69. Mysen, B. O. & Kushiro, I. Compositional variations of co-existing phases with degree of melting of peridotite in the upper mantle. *American Mineralogist* **62**, 843–865 (1977).
70. Takahashi, E. & Kushiro, I. Melting of a dry peridotite at high-pressures and basalt magma genesis. *American Mineralogist* **68**, 859–879 (1983).
71. Asimow, P. D., Hirschmann, M. M., Ghiorso, M. S., O'Hara, M. J., Stolper, E. M. The effect of pressure-induced solid-solid phase transitions on decompression melting of the mantle. *Geochimica and Cosmochimica Acta* **59**, 4489–4506 (1995).
72. Baker, M. B. & Stolper, E. M. Determining the composition of high-pressure mantle melts using diamond aggregates. *Geochimica and Cosmochimica Acta* **58**, 2811–2827 (1994).
73. Baker, M. B., Hirschmann, M. M., Ghiorso, M. S. & Stolper, E. M. Compositions of near-solidus peridotite melts from experiments and thermodynamic calculations. *Nature* **375**, 308–311; 10.1038/375308a0 (1995).
74. Falloon, T. J. & Green, D. H. Anhydrous partial melting of peridotite from 8 to 35 kb and the petrogenesis of MORB. *Journal of Petrology* **29**, 379–414 (1988).
75. Hirschmann, M. M. Mantle solidus: experimental constraints and the effects of peridotite composition. *Geochemistry, Geophysics, Geosystems* **1**, 2000GC000070; 10.1029/2000GC000070 (2000).
76. Hirschmann, M. M., Asimow, P. D., Ghiorso, M. S. & Stolper, E. M. Calculation of peridotite partial melting from thermodynamic models of minerals and melts. III. Controls on isobaric Melt production and the effect of water on melt production. *Journal of Petrology* **40**, 831–851 (1999).
77. Sarafian, E., Gaetani, G. A., Hauri, E. H. & Sarafian, A. R. Experimental constraints on the damp peridotite solidus and oceanic mantle potential temperature. *Science* **355**, 942–945 (2017).
78. Walter, M. J. & Presnall, D. C. Melting behavior of simplified lherzolite in the system CaO–MgO–$Al_2O_3$-$SiO_2$-$Na_2O$ from 7 to 35 kbar. *Journal of Petrology* **35**, 329–359 (1994).
79. Kushiro, I. Partial melting of a fertile mantle peridotite at high pressure: and experimental study using aggregates of diamond. In: Basu, A. & Hart, S. (eds) Earth Processes: Reading the isotopic clock. *Geophysical Monograph, American Geophysical Union* **95**, 109–122 (1996).
80. Falloon, T. J. & Green, D. H. Anhydrous partial melting of MORB pyrolite and other peridotite compositions at 10 kbar: implications for the origin of primitive MORB glasses. *Mineralogy and Petrology* **37**, 181–219 (1987).
81. Green, T. H., Green, D. H. & Ringwood, A. E. The origin of high-alumina basalts and their relationships to quartz tholeiites and alkali basalts. *Earth and Planetary Science Letters* **2**, 41–51 (1967).

82. Maier, W. D., Barnes, S. J., & Karykowski, B. T. A chilled margin of komatiite and Mg-rich basaltic andesite in the western Bushveld Complex, South Africa. Contributions to Mineralogy and Petrology 171, 1–22 (2016).
